# Death from Chloroform

**Published:** 1877-05

**Authors:** 


Death from Chloroform.—Two cases of death from chloroform, administered for anæsthesia in surgical operations, and one to produce sleep, are reported by *British Medical Journal*, as reported by *News and Library*.

Death from this remedy, though rare, occurs sufficiently frequent to awaken some concern for our patients whom we think proper to bring under its anæsthetic influence. Notwithstanding some of the deaths from this cause seem to be the result of a peculiar idiosyncrasy, we have been very much inclined to the opinion that more care in the administration would lessen the number of deaths. It is seldom necessary to induce complete insensibility, and when it is thought advisable to do so, the patient should be gradually brought under the influence, and carefully watched during the process. While some surgeons have almost entirely abandoned the use of chloroform as an anæsthetic, substituting ether, others use it regularly, without seeming to fear any unpleasant or dangerous effects. A mixture of ether and chloroform is used by some, in order to lessen the danger feared from chloroform alone, but after all, may not the idea of greater safety in the use of ether have originated in the fact that fewer deaths are reported, and this without regard to the proportion of cases in which each article was used.

